# Recognizing and engaging pharmacists in global public health in limited resource settings

**DOI:** 10.7189/jogh.09.010318

**Published:** 2019-06

**Authors:** David R Steeb, Rohit Ramaswamy

**Affiliations:** 1 University of North Carolina at Chapel Hill, Eshelman School of Pharmacy, Chapel Hill, North Carolina, USA; 2 University of North Carolina at Chapel Hill, Gillings School of Global Public Health, Chapel Hill, North Carolina, USA

Pharmacists throughout the world are one of the most accessible health care providers and are often the first health professional individuals seek for care. With many pharmacies open 24 hours a day with no appointment necessary, pharmacists are uniquely positioned across communities to provide care and advice, especially for vulnerable populations in limited resource settings. As the pharmacy profession continues to shift from product-centered to patient-centered services such as preventive screenings, immunizations, and disease state management, more attention has been given to the impact pharmacists can have in public health. In developed countries, pharmacists are increasingly recognized and engaged in public health activities such as disease prevention and control programs, policy development, and emergency preparedness among others. Organizations such as the Centers for Disease Control and Prevention (CDC), Public Health England, and the World Health Organization (WHO) are encouraging community organizations and other health care professionals to partner with pharmacists for the effective planning of public health services. In developing countries though, there has not been a comparable endorsement or utilization of pharmacists in public health efforts. With the growing acknowledgement that global health is inclusive of local public health, pharmacists have the opportunity to move the profession forward globally by establishing themselves as health care providers who also serve as global health practitioners.

In limited resource communities throughout the world, there is a particular need and opportunity for pharmacists to be utilized to increase access to safe, quality medications that can save lives. From medication safety and the responsible use of medication to supply chain management and immunizations, pharmacists can utilize their medication expertise not only for addressing local gaps in care, but also for broader global health efforts. Pharmacists in Zimbabwe are providing public health services such as supply chain management, health information provision, and chronic disease state management while community pharmacists in Sudan are providing services such as tobacco cessation, contraception, diet and lifestyle counseling, and obesity and weight reduction advice [1,2].

**Figure:**
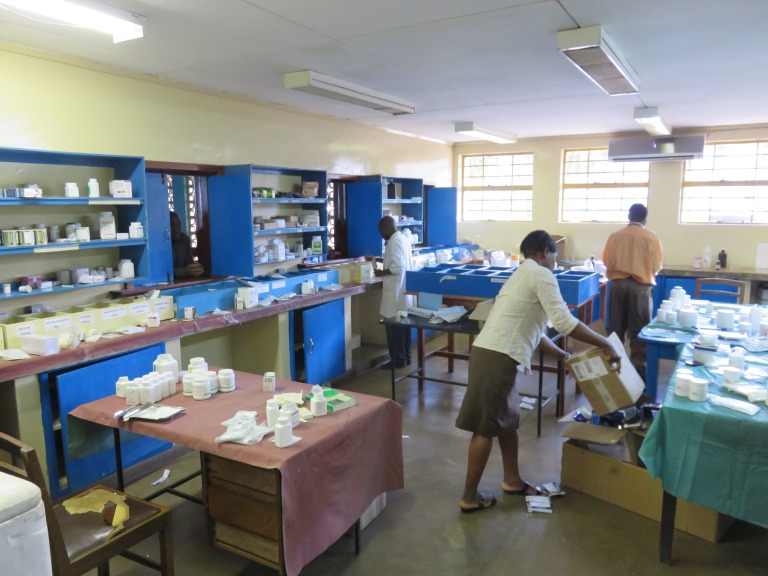
Photo: the Outpatient pharmacy at Kamuzu Central Hospital in Lilongwe, Malawi (from the author’s own collection, used with permission).

Despite a lack of formal recognition, these increased efforts by a few pharmacists in developing countries highlight the emerging opportunity for pharmacists to be engaged in global heath efforts across limited resource settings throughout the world. As the global agenda shifts towards addressing the Sustainable Development Goals (SDGs), pharmacists are primed to collaborate with others to help strengthen pharmaceutical systems in order to achieve the third SDG of good health and well-being for all. Additionally, pharmaceutical systems will need to be resilient to achieve universal health care. As learned from the Ebola crisis, resilient systems function and respond best when there is clarity regarding the roles of different actors within the system including that of pharmacists [3]. The appropriate recognition and engagement of pharmacists in global health efforts across limited resource settings can help systems bounce back and leap forward when challenged. Given that many aims of global health efforts, such as the third SDG, rely upon medications in some capacity, it begs the question as to what role pharmacists can play in helping advance these initiatives. In this paper we describe how pharmacists can utilize their medication expertise in limited resource settings to contribute to not only local public health, but also the global health agenda.

## RATIONAL USE OF MEDICATIONS

Improper medication use is a US$500 billion problem worldwide with pharmacists positioned to be part of the solution given their medication expertise [4]. More than half of this cost is due to medication adherence issues which pharmacists can address through techniques such as motivational interviewing. A recent meta-analysis of self-medication use in developing countries estimates that over 38% of patients self-diagnose and treat contributing to the growing global burden of antimicrobial resistance [5]. Pharmacists can address this by developing optimal treatment regimens based on available resources, providing appropriate patient medication education, and conducting medication reviews to help improve health outcomes. Pharmacists can contribute towards improving medication use and other global health issues at both an individual and systems level. According to an IMS Institute for Healthcare Informatics report on advancing the responsible use of medicines, providing a greater role for pharmacists to own the medication use process was a top five recommendation for ministries of health in order to improve medication use, reduce health care costs, and improve health outcomes [4]. The rational use of medications will be essential in helping address the double disease burden of both communicable and non-communicable diseases in developing countries.

## MEDICATION SAFETY

Substandard and falsified medications pose a significant health risk worldwide with poor quality medications often having the wrong amount or lack of active ingredient leading to increased morbidity and mortality. Developing countries are impacted the most by falsified and substandard medications where life-saving treatments for communicable diseases such as malaria, tuberculosis, and HIV are often the targets. A recent review collectively assessing more than 16 000 samples of medications across Africa, Asia, and South America found that 9%-41% of medications failed to meet quality standards [6]. Medication errors also pose significant issues to medication safety. The WHO recently launched the third iteration of the Global Patient Safety Challenge with a focus on reducing avoidable harm caused by medications over the next five years, which positions pharmacists to have a significant impact. Given their role in quality assurance across pharmaceutical systems, pharmacists can contribute to the design of processes and programs to mitigate medication errors, improve medication quality, and improve health outcomes across a variety of health care settings. From manufacturing roles within the pharmaceutical industry to the community pharmacy where innovative approaches are being developed to test medication quality on demand, pharmacists can impact the health of populations in different ways. Beyond quality assurance and medication errors, pharmacovigilance, or the detection and prevention of adverse drug reactions, is another aspect of medication safety for pharmacist engagement.

## SUPPLY CHAIN MANAGEMENT

Effective supply chain management ensures access to essential medications and is a key factor for achieving the sustainable development goal of universal health coverage. It is estimated that fewer than 30% of patients in the WHO Africa region have public access to essential medications [7]. While the rational use of medications is an important issue, addressing this depends on patient and government access to what is needed. The WHO’s “Towards Access 2030” framework seeks to strengthen pharmaceutical systems as medications often constitute the largest portion of health spending at both a country and individual level [8]. The framework targets developing countries who often lack the technical, financial, and governance capacity to ensure affordable access to quality medications. Pharmacists can engage within this framework and pharmaceutical systems to form and evolve national essential medication lists in accordance to community needs and guideline recommendations. Pharmacy professionals have been successfully utilized in programs such as the Accredited Drug Dispensing Outlet (ADDO) initiative in Tanzania, which has increased the adoption of treatment guidelines, led to significantly higher availability of essential medications for children, and increased quality of dispensed medications [9]. The ADDO program, which has been adapted across other countries including Uganda, Liberia, and Zambia, could serve as model for implementation in other limited resource settings throughout the world.

## RECOMMENDATIONS FOR IMPROVING ENGAGEMENT AND UTILIZATION OF PHARMACISTS

While there are factors associated with each of these areas that play a role in determining outcomes, the engagement and utilization of pharmacists can be an initial step towards advancement. Limited resource settings have several complex barriers though that affect the ability of pharmacists to engage further in a global health context. Factors known to hinder public health services offered by pharmacists in developing countries include a lack of knowledge and skills, lack of confidence, limited policies and regulations, poor recognition within the health care system, inadequate workforce capacity, and patient reluctance to use pharmacy services [10]. While these factors are multifaceted and complex, the following recommendations are provided for initial consideration as to how to best engage pharmacists in global health across limited resource settings:

### 1) Recognize and utilize pharmacists as part of the public health workforce

As done in developed countries, formal recognition of pharmacists by associations and governments in developing countries as part of the public health workforce can provide the confidence needed for pharmacists to act in a global health context. With the continued discussion of the global health workforce shortage and the lack of appropriately trained providers in limited resource settings, maximizing the skills and knowledge of pharmacists can fill local gaps in care and provide a platform for different levels of professional development including global health engagement. The above-suggested areas of the rational use of medications, medication safety, and supply chain management are areas governments and organizations should explore further as to the role of the pharmacist. Those pharmacists already engaged in these efforts should be recognized for their work contributing to global health efforts such as the SDGs. Pharmacists are particularly ready to help address the epidemic of non-communicable diseases given the importance of long-term medication regimens to manage chronic disease states. With their medication knowledge and community accessibility, pharmacists can also engage in recommending lifestyle modifications and preventative measures. To do so, pharmacists must develop collaborative relationships with not only other members of the health care team, but also the community. Increased utilization of pharmacists leveraging their medication expertise and engaging them in the community can allow for higher job satisfaction and decreased attrition to strengthen local capacity for workforce development.

### 2) Incorporate global public health principles in pharmacy education

Whiie pharmacists have the technical skills and competencies based on medication expertise to contribute to global health efforts, they often lack the foundational public health knowledge needed to maximize themselves as global health practitioners. In order to implement and scale public health services, students and pharmacists should be appropriately trained in basic public health competencies to better be able to understand how patients fit into populations and how to effectively manage the health of a community. This model has been successfully incorporated in the accreditation standards of pharmacy schools across several developed countries, including the United States and the United Kingdom, and has the potential to lead to change in other settings as well. In addition to foundational public health principles, students and pharmacists in developing countries should be trained in the basics of program planning, quality improvement, implementation, and monitoring and evaluation. This skill set will equip graduates with the capability of putting research into practice and to scale-up services. The complementary knowledge and skills in public health can empower pharmacists to serve as global health leaders in their community and abroad.

### 3) Utilize implementation science methods in pharmacy across limited resource settings

Many of the above mentioned challenges in low resource settings have potential solutions that have been utilized in high-income countries. However, it is unknown how evidence based practices from the global north translate to the global south. Implementation science is the field of understanding how to translate evidence into everyday practice and can be utilized across public health issues from medication safety to mobile health. While implementation science is stimulating research opportunities in the community and primary care settings for pharmacy in the United States, there is a lack of research regarding the effective implementation and evaluation of pharmacy services within developing countries. There is a need for the appropriate monitoring and evaluation of pharmacy based public health services so that these can be adopted and scaled up accordingly. Implementation research for strengthening pharmacy systems and delivery processes in low to middle income countries can help provide the data needed for government leaders to support the integration of pharmacists into public health services. As pharmacists develop their niche within global public health, there will be the need to understand how and why pharmacy services can improve the health and well-being of communities throughout the world in resource limited settings.

## CONCLUSION

Pharmaceutical systems across limited resource settings worldwide need to be strengthened in order to achieve the broader goals of the global health agenda. Pharmacists as the medication experts can contribute to key global health areas to strengthen these systems across different levels, but action must be taken to enable pharmacists to serve in this capacity. As pharmacists gain recognition of their role contributing to the health of communities, there will be expanded opportunities for pharmacists to help reduce health disparities and achieve good health for all.
